# Implementation outcomes of cognitive behavioural therapy delivered by non-specialists for common mental disorders and substance-use disorders in low- and middle-income countries: a systematic review

**DOI:** 10.1186/s13033-020-00372-9

**Published:** 2020-05-29

**Authors:** Ibone J. Verhey, Grace K. Ryan, Nathaniel Scherer, Jessica F. Magidson

**Affiliations:** 1 Centre for Global Mental Health, London School of Hygiene and Tropical Medicine (LSHTM), Keppel Street, London, WC1E 7HT UK; 2 Department of Psychology, University of Maryland, College Park, MD USA

**Keywords:** Global mental health, Cognitive behavioural therapy, Non-specialist health workers, Common mental disorders, Substance-use disorders

## Abstract

Due to severe shortages of specialist mental health personnel in low- and middle-income countries (LMICs), psychological therapies are increasingly being delivered by non-specialist health workers (NSHWs). Previous reviews have investigated the effectiveness of NSHW-delivered psychological therapies, including cognitive behavioural therapy (CBT), in LMIC settings. This systematic review aims to synthesise findings on the implementation outcomes of NSHW-delivered CBT interventions addressing common mental disorders and substance-use disorders in LMICs. Four databases were searched, yielding 3211 records, 18 of which met all inclusion criteria. We extracted and synthesised qualitative and quantitative data across eight implementation outcomes: acceptability, adoption, appropriateness, feasibility, fidelity, implementation cost, penetration and sustainability. Findings suggest that delivery of CBT-based interventions by NSHWs can be acceptable, appropriate and feasible in LMIC settings. However, more research is needed to better evaluate these and other under-reported implementation outcomes.

## Introduction

Cognitive behavioural therapy (CBT) is an effective psychological intervention for the treatment of common mental disorders (CMD) and substance use disorders (SUD) that is widely used in high-income countries [43]. In low- and middle-income countries (LMICs), where specialist mental health care providers are often scarce, there has recently been a focus on developing CBT-based interventions for delivery by non-specialist health workers (NSHWs) [1, 6, 23, 32]. A NSHW is defined as a health worker who does not have specialised training in mental health, but can deliver interventions under the supervision and training of more specialised providers [44]. NSHWs can increase the availability and accessibility of mental health care, particularly at the community-level [44]; however, the interventions that NSHWs are expected to deliver should be straightforward and brief, and must be accompanied by regular training and supervision to ensure fidelity to the intervention package [11, 24].

Recent reviews have mainly investigated the effectiveness of NSHW-delivered psychological interventions (including CBT) in terms of patient outcomes. For example, a systematic review and meta-analysis of randomised controlled trials (RCTs) conducted in LMICs concluded that NSHW-delivered psychological interventions are effective in reducing symptoms of anxiety, depression and post-traumatic stress disorder [39]. A Cochrane review also examined the effectiveness of mental health interventions—including psychological interventions—when delivered by NSHWs [44]. Neither of these reviews included SUD. Previous reviews have not synthesised the evidence on implementation outcomes of NSHW-delivered CBT in LMICs. Implementation outcomes are defined as “the effects of deliberate and purposive actions to implement new treatments, practices and services” [37]. A popular taxonomy of implementation outcomes developed by Proctor et al. [37] differentiates between patient, service and implementation outcomes, and divides the latter into the following categories: acceptability, adoption, appropriateness, cost, feasibility, fidelity, penetration and sustainability [37]. Evaluating these implementation outcomes can provide important insights into how a treatment achieves (or fails to achieve) its effects, including the barriers and facilitators to effective service delivery [9, 15].

The primary objective of this review is to address key gaps in the literature, in three ways, by: (1) focusing primarily on implementation outcomes, complementing prior systematic reviews focused on effectiveness outcomes, to better understand how CBT interventions are delivered by NSHWs; (2) extending the search beyond RCTs to include pilot, feasibility and qualitative studies, which often report on key aspects of implementation; and (3) including SUD, which are prevalent in LMICs and can be treated with CBT but were not included in prior systematic reviews on CBT in LMICs. The secondary objectives are: (1) to explore implementation outcomes by provider type (lay health worker, lay counsellor, peer or paraprofessional); (2) to examine how provider-level factors facilitate implementation; and (3) to identify how training and supervision strategies were used to support implementation.

## Methods

The search strategy was developed following a brief scoping review to identify key domains and sources of literature used in previous reviews on similar topics [4, 17, 22, 29, 38, 44, 45]. To allow for the inclusion of heterogenous studies, methods of narrative synthesis were selected [19, 36]. Narrative synthesis refers to a systematic approach to address a range of questions (in lieu of or in addition to questions regarding effectiveness), by “relying primarily on the use of words and text to summarise and explain the findings” [36]. For the purposes of this synthesis, the implementation outcome variables framework by Proctor et al. [37] served as a framework for data extraction and analysis [37]. The protocol for the review was published on Prospero (CRD42018100087), and further details on methods are provided below.

### Search strategy and selection criteria

The search covered four domains: (1) Low- and middle-income countries (2) cognitive behavioural therapy (3) non-specialist health workers and (4) Mental, neurological and substance-use disorders. Subject headings and search terms were adapted from a similar review [38] for Ovid MEDLINE(R) (1946–2018), Embase (1974–2018), PsycINFO (1806–2018) and Global Health (1910–2018) (see Appendix). Forward and backward citation searches were also conducted.

Studies were considered for inclusion if they: (1) were carried out in a LMIC (as per the World Bank classification during the year of publication); (2) investigated a CBT-based intervention (see below); (3) were delivered by a NSHW (see Table 1); (4) addressed a CMD/SUD (as per the International Classification of Diseases [ICD-10]); and (5) reported on one or more of the eight implementation outcomes identified by Proctor et al. [37] (either quantitatively or qualitatively). Based on Tolin’s [43] recent review, CBT-based interventions were defined as psychological interventions involving one or more of the following CBT components: relaxation training, behavioural rehearsal (including problem solving), exposure therapy, cognitive restructuring and/or operant procedures (including behavioural activation). No control group was required in order to be considered for inclusion.

**Table 1:** Types of NSHWs in included records

Type of NSHW	Definition	Trained & supervised by:	Study	Author (year)
Lay health workers (e.g. community health workers or Lady Health Workers)	Non-specialist workers linked to the local health system (part of formal health workforce) Living locally; often mobile in the community Empathy, interpersonal skills, motivation Gatekeepers to the community for new interventions	Senior health promotion officers or Mental health specialists	Friendship Bench Programme	Chibanda (2016) Chibanda (2017)
			Problem-solving therapy	Munodawafa (2017) Nyatsanza (2016)
Lay counsellors (e.g. lay-helpers)	Selection based on competency assessment (delivery & skills) Intensive supervision needed	Lay health workers or Mental health specialists	Common Elements Treatment Approach	Murray (2014) Bolton (2014)
			Healthy Activity Programme	Chowdhary (2016)
			Trauma-focused CBT vs. Problem-solving therapy	Dawson (2018)
			Problem Management Plus	Khan (2017)
			Counselling for Alcohol Problems	Nadkarni (2015) Nadkarni (2017)
Peers	Similar lived experience as service users Age, gender and language matching as facilitators Less formal boundaries/more flexibility in delivery	Non-specialist facilitators	Thinking Healthy Peer	Atif (2016) Atif (2017) Singla (2014)
Paraprofessional counsellors	Little or no background in counselling or psychology Trained and supervised to deliver manualised therapy	Mental health specialists	Cognitive processing therapy	Bass (2013)
			Group intervention	Tol (2008)
			Culturally adapted group CBT	Papas (2010) Papas (2011)

Studies were excluded if they (1) were set in high-income countries; (2) did not include at least one CBT component; (3) were not delivered by a NSHW; (4) did not address CMD/SUD; or (5) did not report on at least one implementation outcome.

### Screening, quality assessment and data extraction

The second reviewer (NS) double-screened 20% of all records, first at the stage of title and abstract screening, then at full-text screening. NS also performed an independent quality assessment of the included studies. Discrepancies were resolved through discussion between the two reviewers (NS and IV).

Qualitative studies were assessed with the Critical Appraisal Skills Programme (CASP) checklist [14]. Randomised studies were assessed with the Cochrane Collaboration tool for assessing the risk of bias [20]. Non-randomised studies were assessed with the Cochrane Risk Of Bias in Non-randomised Studies-of Interventions (ROBINS-I) [13, 41]. Overall risk of bias ratings were assigned as follows: low risk, when all/most of the criteria of the checklists were fulfilled (50% of studies); moderate risk, when some criteria were fulfilled (38.9%); or high risk, when few or no criteria were fulfilled (11.1%) [25].

Using a data extraction sheet developed for this review, data were entered into Excel 2018 and then imported into NVivo 12 for qualitative coding by IV

## Results

**Fig. 1:**
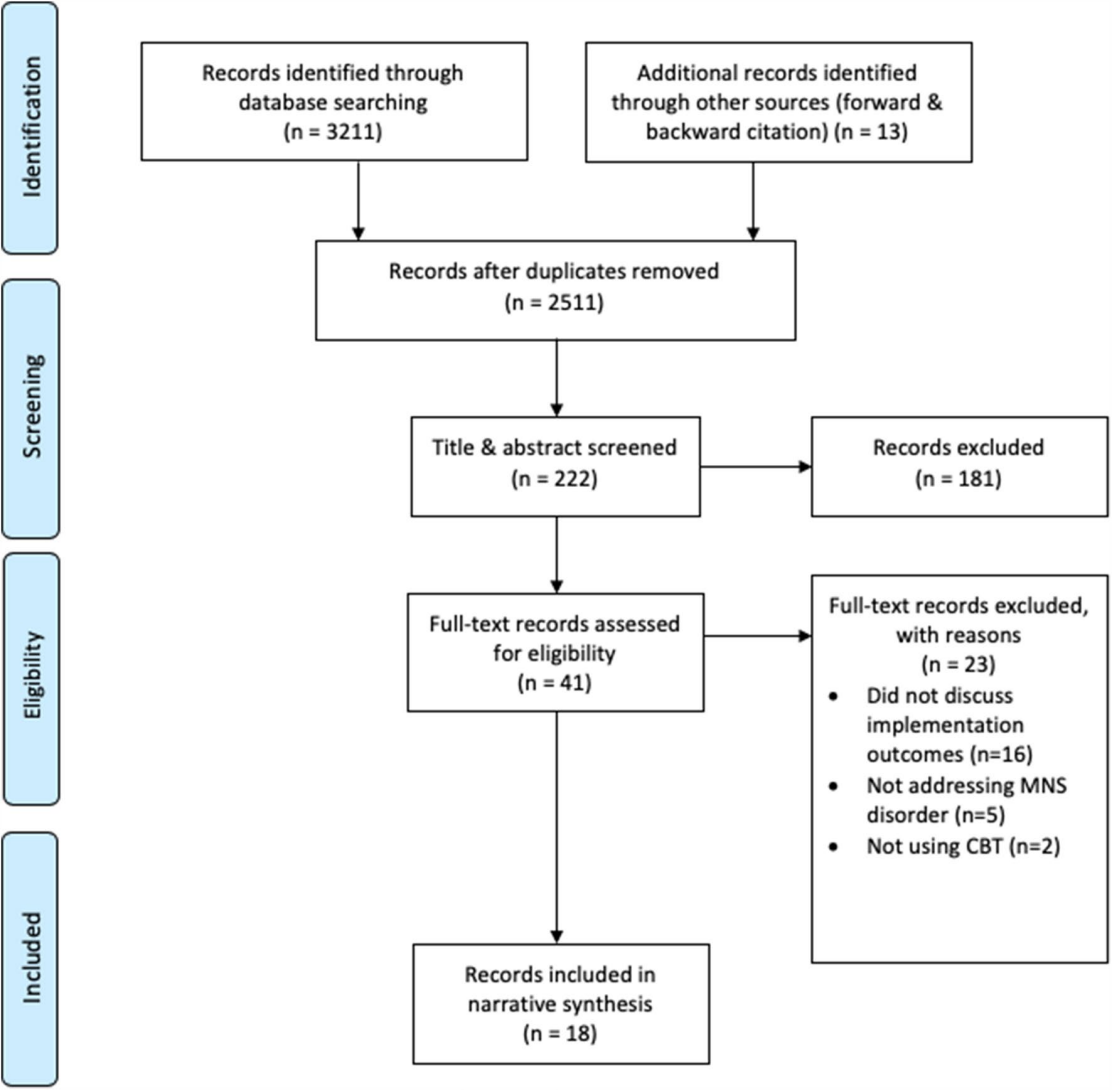
Study selection Adapted from the PRISMA Group (Liberati 2009)

A total of 3211 records were identified through database searching and 13 records through forward and backward citation searches. After duplicates were removed, the remaining 2511 were title and abstract screened, and 41 were considered for the full-text stage. 18 records assessing 11 distinct studies carried out in nine countries (India, Pakistan, Zimbabwe, Kenya, Indonesia, Democratic Republic of Congo, South Africa, Thailand and Iraq) met all of the criteria for inclusion (see Fig. 1). As some of the records assessed the different parts of the same intervention, the unit of analysis for this paper will be the distinct studies to avoid skewing the findings towards studies with multiple records (see Table 2). There was no date restriction in our search, but all included studies were conducted between 2008 and 2018. The CBT components used in the 11 studies (see Table 2 for details) were: problem-solving therapy (64%), behavioural activation and cognitive restructuring (55% each), exposure therapy (27%) and relaxation (18%). The interventions were delivered by lay health workers (27%), lay counsellors (55%), peers (9%) and paraprofessional counsellors (27%) (see Table 1 for details).

**Table 2:** Study characteristics (18 records from 11 distinct studies)

Study	Author (year)	Country	Setting	Total study population/ gender	Study Design	Disorder targeted	Type of NSHW/ gender
Treatment components							
***Thinking Healthy Programme Peer*** – Behavioural activation – Problem-solving therapy	Atif (2016)	Pakistan	Primary healthcare	49 Female	Qualitative	Perinatal depression	Peers Female
	Atif (2017)	Pakistan & India	Primary healthcare	102 Individual interviews & 15 Focus group discussions Female	Qualitative	Perinatal depression	Peers Female
	Singla (2014)	Pakistan & India	Primary healthcare	99 Individual interviews & 13 Focus group discussions Female	Qualitative	Perinatal depression	Peers Female
***Cognitive processing therapy***	Bass (2013)	Democratic Republic of Congo	Community-based	405 Female	Randomised Controlled Trial (RCT)	Depression, anxiety, post-traumatic stress disorder (PTSD)	Para-professionals Mixed
***Common elements treatment approach*** – Relaxation – Behavioural activation – Cognitive restructuring – In vivo exposure – Motivational interviewing	Bolton (2014)	Thailand (Burmese refugees)	Community-based	437 Mixed	RCT	Depression, anxiety, PTSD	Lay-counsellors Mixed
	Murray (2014)	Thailand & Iraq	Community-based	34 Mixed	Pilot RCT	Depression, anxiety, PTSD	Lay-counsellors Mixed
***Friendship bench programme*** – Problem-solving therapy	Chibanda (2016)	Zimbabwe	Primary healthcare	573 Mixed	RCT	Depression & anxiety	Lay health workers Female
	Chibanda (2017)	Zimbabwe	Primary healthcare	17 Mixed	Qualitative	Common mental disorders (CMD)	Lay health workers Female
***Healthy activity programme*** – Behavioural activation – Problem-solving therapy – Relaxation training	Chowdhary (2016)	India	Primary healthcare	55 Mixed	Pilot RCT	Severe depression	Lay-counsellors Mixed
***Trauma-focused CBT vs. Problem-solving therapy*** – Cognitive restructuring – In vivo exposure	Dawson (2018)	Indonesia	School-based	64 Mixed	RCT	PTSD (children)	Lay-counsellors Mixed
***Problem Management Plus*** – Behavioural activation – Problem-solving therapy	Khan (2017)	Pakistan	Community-based	119 Mixed	Cluster pilot RCT	CMD	Lay-helpers Mixed
***Problem-solving therapy*** – Behavioural activation – Healthy thinking	Munodawafa (2017)	South Africa	Primary healthcare	6 Female	Qualitative	Perinatal depression	Community health workers Female
	Nyatsanza (2016)	South Africa	Primary healthcare	26 Female	Qualitative	Perinatal depression	Lay health workers Female
***Counselling for Alcohol Problems*** – Cognitive skills (handling of difficult emotions) – Problem-solving therapy – Drink refusal skills – Motivational interviewing	Nadkarni (2015)	India	Primary healthcare	53 Male	Pilot RCT and Qualitative	Alcohol use disorder (AUD)	Lay-counsellors Mixed
	Nadkarni (2017)	India	Primary healthcare	377 Male	RCT	AUD	Lay-counsellors Mixed
***Culturally adapted group CBT*** – Drink refusal skills – Problem-solving therapy – Cognitive restructuring	Papas (2010)	Kenya	Primary healthcare	27 Mixed	Pilot feasibility study	AUD	Para-professional counsellors Mixed
	Papas (2011)	Kenya	Primary healthcare	75 Mixed	Pilot RCT	AUD	Para-professional counsellors Mixed
***Group intervention*** – CBT techniques with cooperative play and creative activities – Trauma processing	Tol (2008)	Indonesia	School-based	495 Mixed	Cluster RCT	PSTD & Anxiety (children)	Para-professionals Mixed

Of the 11 studies, seven focused on appropriateness (64%), seven on feasibility (64%), six on acceptability (54%) and six on fidelity (54%), followed by three studies focusing on adoption (27%), two on sustainability (18%), one on penetration (9%) and one on implementation cost (9%) (see Table 3 for details). The definitions of the implementation outcomes were based on Proctor’s model [37] (see Table 4 for definitions); however, these outcomes were assessed using a variety of methods.

**Table 3:** Implementation outcomes discussed in included records (n = 18)

Study	Author (year)	Acceptability	Adoption	Appropriateness	Feasibility	Fidelity	Impl. Cost	Penetration	Sustainability
Thinking Healthy Peer	Atif (2016)	✓		✓					✓
	Atif (2017)	✓	✓	✓	✓				✓
	Singla (2014)	✓	✓	✓	✓				
Cognitive processing therapy	Bass (2013)			✓		✓			
Common Elements Treatment Approach	Bolton (2014)	✓		✓	✓	✓			
	Murray (2014)	✓		✓	✓	✓			
Friendship Bench Programme	Chibanda (2016)	✓	✓	✓		✓			✓
	Chibanda (2017)	✓		✓		✓		✓	✓
Healthy Activity Programme	Chowdhary (2016)			✓	✓				
Trauma-focused CBT vs. Problem-solving therapy	Dawson (2018)					✓			
Problem Management Plus	Khan (2017)	✓	✓		✓				
Problem-solving therapy	Munodawafa (2017)	✓		✓		✓			
	Nyatsanza (2016)	✓		✓	✓	✓			
Counselling for Alcohol Problems	Nadkarni (2015)	✓							
	Nadkarni (2017)	✓				✓	✓		
Culturally adapted group CBT	Papas (2010)			✓	✓				
	Papas (2011)				✓	✓			
Group intervention	Tol (2008)					✓

**Table 4:** Implementation research outcome framework Based on Proctor et al. [37]

Implementation outcome	Definition (Proctor et al. 2011)	Example from included studies
Acceptability	The perception of stakeholders that the intervention is agreeable or satisfactory	Peers seen as acceptable providers for the ‘Thinking Healthy Programme’ by service users due to their similar experience and interpersonal skills (Atif 2016)
Adoption	The process of putting an intervention to use	Adoption was facilitated by perceived usefulness of ‘Problem Management Plus’ to service users, providers and the community (Khan 2017)
Appropriateness	The fit, relevance or compatibility of the intervention for the setting, service provider or service user	CBT components were appropriate as part of the ‘Common Elements Treatment Approach’ as they reflected cultural practices among the Burmese refugees receiving the intervention (Bolton 2014)
Feasibility	The extent to which an intervention can be successfully carried out	The “highly structured format” of CBT was found to make it feasible for delivery by paraprofessionals (Papas 2010)
Fidelity	The extent to which the intervention was implemented according to its original design	Motivated, well-trained lay health workers followed the manual more closely to deliver the intervention as intended (Munodawafa 2017)
Implementation cost	The overall cost of delivery of an intervention	An economic assessment suggests that ‘Counselling for Alcohol Problems’ is likely to be cost-effective in terms of recovery from alcohol-use disorders (Nadkarni 2017)
Penetration	The integration of the intervention into a routine service or a measure of how many those eligible are receiving it	‘The Friendship Bench’ has provided care to 7000 individuals between 2006 and 2011 (Chibanda 2017)
Sustainability	The maintenance of an intervention and its continued use	The lay health workers have continued to deliver the intervention following the completion on the randomised trial (Chibanda 2017)

### Synthesis

#### Key barriers and facilitators across implementation outcomes

*Appropriateness* The seven studies that reported appropriateness outcomes indicated that using a relevant local language, culturally appropriate terms and colloquial expressions increased local recognition for the appropriateness of the intervention [2, 3, 7, 8, 10–12, 24, 27, 33, 35]. One study specifically reported that CBT already reflected some of the cultural practices among the Burmese service users, such as meditation (relaxation); harmony, positive thinking (cognitive restructuring) and reengaging with traditional activities (behavioural activation) [7].

*Feasibility* The use of CBT components was shown to be feasible for NSHW delivery by seven studies [2, 3, 7, 10, 12, 24, 27, 30, 33, 40]. The selection of feasible strategies and components was based on piloting/feasibility studies (*n *= 4) or qualitative studies (*n *= 3) involving stakeholders. Facilitators to increase feasibility were perception of usefulness by providers and service users, a context-specific intervention and standardised steps for simpler decision-making and delivery [2, 7, 12]. While Chibanda et al. [11] and Dawson et al. [16] expressed concern about the complexity of the cognitive component of standard CBT, Papas et al. [34, 35] suggested that its “highly structured format” made it feasible for delivery by trained NSHWs, especially when shortened from 12 to six sessions.

*Acceptability* Five studies reported that acceptable interventions contained beneficial information and skills for the service user and the community [2, 3, 11, 12, 24, 31]. Some barriers to acceptability were women’s lack of autonomy (both as service users and providers), cultural barriers, stigma, lack of engagement and resistance to changes in sociocultural hierarchies [3, 33]. Some service users reported concerns about whether their confidentiality would be ensured, especially in group interventions or if there were family members present [2, 24]. A study assessing an intervention with two study locations reported differences in the acceptability of having mixed-gender provider-service user pairs; in Thailand, this was acceptable, while in Iraq a third counsellor had to be present [7].

*Fidelity* Culturally appropriate language, simplified interventions and supporting materials were considered useful strategies to improve fidelity and understanding [10, 12, 16]. Seven studies reported that clear manuals, training and weekly supervision were ways to increase fidelity to the intervention by providing opportunities for feedback and supporting with difficult cases [2, 5, 7, 12, 16, 24, 27]. One study reported that training helped improve fidelity and that this was especially relevant to NSHWs who had previously been responsible for health promotion, as a shift from advice-giving to service user-led problem solving therapy was required [27].

*Adoption* Engaging families and financial remuneration were motivating factors for the peer volunteers to promote adoption of CBT [2, 3, 40]. A barrier to adoption was lack of perceived usefulness of counselling by the communities [3, 11, 33]. A facilitator to adoption was the perceived usefulness of the intervention to service users, providers and the community [2, 24].

*Sustainability* The sustainability of one program was strengthened through integration into routine clinical practice [10, 11] and high levels of motivation and suitable supervision and incentivisation were found to be further facilitators [3].

*Penetration* Aside from the total number of individuals reached during the course of the trial, penetration was not addressed by the majority of studies. One study measured the total population reached after the intervention was integrated into routine practice [10].

*Implementation cost* was not assessed by any of the studies, although one did compare the cost effectiveness of the intervention to enhanced usual care [31].

### Secondary objectives

#### Implementation outcomes by provider type

Implementation outcomes were found to differ based on provider type (lay health worker, lay counsellor, peer or paraprofessional). Lay health workers were employed by the government as part of a formal health workforce [2, 11, 24], which was both a benefit due to the strong links to the local health system and high fidelity of delivery, and a challenge due to potential overburdening [2, 40]. Using pre-existing lay health workers and their supervision systems as a basis for new interventions, made this a feasible and sustainable choice of NSHW [10, 40]. Lay health workers were acceptable to stakeholders, respected in the community and well-integrated [2, 3, 10, 11, 27, 40].

The five studies assessing programmes that used lay-counsellors highlighted the importance of choosing acceptable components and appropriate strategies, recruiting locally and using simplified and relevant language for feasibility and fidelity of delivery [7, 12, 16, 24, 31]. Four of the studies discussed fidelity, reporting good adherence to interventions that had been tailored appropriately for delivery by lay-counsellors [7, 16, 24, 30, 31]. All five discussed the importance of comprehensive training and intensive supervision to ensure competency [7, 12, 16, 24, 31].

In India and Pakistan, the Thinking Healthy Programme transitioned service delivery from lay health workers to peer volunteers, reducing implementation costs via a more feasible reimbursement system (compared to pre-specified health worker salary schemes) [40]. Peers were considered acceptable due to their personal experience and ability to connect with users [2, 3, 40].

Paraprofessionals were found have moderate to high fidelity when supported by a specifically adapted manual [8, 34, 35, 42]. The selection process was based on skills and competencies as evidenced by written or practical work [8, 34, 35, 42].

#### Provider-level factors and implementation outcomes

Provider-level factors may directly influence implementation, which could help contextualise the differences in intervention outcomes. Five studies discussed NSHW motivation as a factor influencing and impacted by acceptability [2, 3, 7, 10, 11, 27, 30, 33, 40]. Acceptability of NSHWs came partly from their personal characteristics, such as living in the same community, having good communication skills, being experienced and being able to form relationships [2, 3, 7, 27, 33, 34, 40]. Two studies reported that community acceptability of the intervention was increased through the use of well-trained and incentivised peer providers who were linked with the health care system [3, 10]. NSHW motivation was influenced by personal gain, support from the community, positive feedback from service users and enhanced social standing, as well as opportunities to learn new skills and gain experience [2, 3, 27, 30]. Female providers were chosen to deliver interventions to women with perinatal depression in India, Pakistan and South Africa due to their ability to relate to this service user group [27, 40] and, in Zimbabwe, older women were chosen as they were respected and trusted in the community [10, 11]. In addition to being peers, the providers were expected to be of the same religion and a higher social standing than the service user in rural Pakistan, while in urban Goa (India) an individual’s standing within her family was seen as more relevant than caste or religion [40]. The intervention assessed by Nadkarni et al. [30] targeted only men, as alcohol use disorders have been identified as a leading cause of disease burden among this service user group (WHO, 2014), however, mixed gender providers were trained to deliver it. All other studies assessed interventions delivered by mixed gender providers, including the two studies assessing school-based interventions addressing children’s mental health [16, 42], as they were addressing common mental disorders affecting people of all genders. Intervention delivery location varied across context; five studies reported acceptability of home-based delivery and/or follow-up sessions [2, 7, 11, 12, 30, 31, 40], while service users in South Africa preferred clinic-based sessions due to confidentiality and safety [27, 33].

#### Training and supervision strategies

Effective training and supervision were found to be key components of successful implementation. Practical training provided learning and skill-building opportunities, as well as sustaining motivation [2, 16, 24, 27]. The length and type of training provided to the NSHWs depended on the intervention and on practical issues. Four studies found that task-specific training followed by a period of practical work was beneficial in preparing NSHWs for intervention delivery [12, 16, 24, 31]. Team meetings during training, which later became the supervision groups, created a supervision system for NSHWs that took part in two of the interventions and allowed for peer learning and problem-solving [16, 24]. The use of pre-existing NSHWs as training providers, support systems or as supervisors helped to create a sustainable system for new interventions, as well as making use of the knowledge and experience of the existing NSHWs [10, 24].

Supervision strategies differed by intervention according to the needs of the NSHWs and feasibility of delivery. Supervision was delivered in groups and/or individually by peers, pre-existing NSHWs or specialists. Some interventions had face-to-face supervision in place, while others utilised web-based or telephone-based systems. Peers were the NSHWs most in need of supervision as they had the least experience delivering interventions; they received individual face-to-face “emotional and practical support” from their supervisors, which helped to sustain fidelity and motivation and increase their credibility in the community [2]. Fidelity was high among lay-counsellors who received web-based supervision from specialists abroad, and although this was not more time-intensive, it may not be a feasible or sustainable system [7, 16, 24, 26]. Close supervision helped to maintain fidelity to the intervention manual, provided support for the NSHWs and opportunities to monitor the quality of delivery, for example through assessing randomised video tapes of sessions [10, 16, 34].

Different models discussed in the studies were the cascade model, in which specialists train facilitators who in turn train the front-line providers [3], and the apprenticeship model, which provides on-the-job training [24, 28]. The former approach may take pressure off specialists, allowing for further task-sharing and widespread training, while the latter can ensure comprehensive hands-on training and integration. Multi-tiered supervision systems, like the apprenticeship model, allow for further task-sharing and provide feedback loops to increase the appropriateness of the intervention, integrate NSHW input and monitor fidelity [7, 11, 26, 27].

## Discussion

The findings from our systematic review suggest that implementation was feasible when the intervention used language, methods and providers that were culturally appropriate and acceptable to the target population. Cultural values, norms and expectations can impact stakeholder buy-in, acceptability, adoption and penetration of the program, and ultimately its sustainability [8]. Basing adaptations on the setting is especially relevant for interventions, like CBT, that were originally developed for specialist-delivery in high-income countries [7, 23, 26]. The included studies reported that feasibility testing and qualitative research were used to guide adaptations to CBT interventions, choice of NSHW and delivery methods.

The NSHWs were chosen based on their ability to relate to the service user group, due to their standing in the community and other personal characteristics, such as good communication and interpersonal skills. Gender matching of provider-service user pairs took place when relevant to the nature of the intervention and for cultural reasons, but most studies assessed interventions where mixed- gender providers addressed mixed-gender service user groups. Similarly, the nature of the intervention impacted the location of intervention delivery, for example in school, community or primary healthcare, as well as the appropriateness of home visits. The latter was also influenced by cultural issues, safety and confidentiality concerns [2, 24]. The development of a given intervention depended in part on the service user category and the mental disorders being addressed, which in turn influenced the type of NSHW chosen to deliver it and the adaptations needed to make a CBT-based intervention feasible and appropriate for this category. The findings suggest that, to increase the acceptability and appropriateness of the intervention, cultural issues surrounding mental disorders should be taken into account, as well as a relevant language and culturally appropriate terms used [10, 12, 16].

The findings reflect the appropriateness of CBT-based interventions for the given service user groups, but contextual adaptations were needed to ensure that the language used and the form of delivery was acceptable and appropriate to both service users and providers. Papas et al. [35] used a cultural adaptation framework incorporating concepts, language and context to guide the adaptations and test their feasibility. Tools used for diagnosis and screening should similarly be adapted and validated, or they may be unable to measure psychological distress in that setting or impose an inappropriate treatment [7, 8, 11, 16, 42].

Research evaluating task-sharing of psychological interventions typically lacks differentiation across distinct types of NSHWs, and few prior systematic reviews have sought to compare outcomes by these types or to synthesise the training and supervision strategies of the interventions. Findings from this review suggest that the choice of NSHW may have an impact on fidelity to delivery and the acceptability, feasibility and sustainability of the intervention. As highlighted previously, there are important differences among NSHWs utilised in task sharing models, and each type may uniquely affect implementation outcomes. As NSHWs rarely have prior experience of working in mental health, the intervention should be brief and straightforward, and CBT may be a particularly good fit given its structured format for training and delivery [35]. Future research should continue to take a more nuanced approach to understanding how different types of providers in task sharing models may affect important implementation outcomes, which in turn may also influence effectiveness outcomes.

Provider-level factors that facilitated implementation included the motivation and skills of the NSHWs, the acceptability and appropriateness of the type of NSHW and their relationship with the service users. NSHW motivation influenced various implementation outcomes, including acceptability to the community and fidelity to the intervention. Motivation was influenced by perceived opportunities to learn and gain experience, extend social networks, and increase career chances and income, in addition to community endorsement and positive feedback [2, 3, 7, 10, 11, 27, 30, 33, 40]. Key factors included communication and interpersonal skills, perceived wisdom and standing in the community, in addition to the NSHWs’ proximity to the service users. The findings reflected the importance of having a motivated cadre of NSHWs who are accepted and trusted to deliver an appropriate intervention that is perceived as useful by the community.

There was high fidelity to, acceptability and feasibility of manualised interventions that were developed and adapted for the context, based on preliminary work involving stakeholders, and supported by rigorous training and frequent supervision. A suitable training method was found to be important in order to engage stakeholders, maintain fidelity and facilitate recruitment and retention of NSHWs [2, 7, 8, 12, 27]. During supervision, NSHWs were able to address challenges and receive feedback and support [2, 7, 33, 34]. Peers needed high levels of training, supervision and ongoing support due to their personal involvement in the intervention, while some lay health workers worked within pre-existing training and supervision systems. Paraprofessionals and lay counsellors needed varying degrees of training, supervision, and consultation when there was a lack of fidelity or other challenges. In low-resource settings in high income countries, telephone- and web-based forms of supervision have also shown good outcomes for provider training, supervision and support and may continue to be feasible solution to reaching more remote populations [21]. Whether it took place virtually or face-to-face, adequate training and intensive supervision helped ensure that interventions could be delivered by NSHWs with high fidelity to the manual guidelines, while providing a system of accountability and a way to monitor the implementation of the intervention.

## Limitations

To allow for inclusion of heterogeneous implementation outcomes and mixed methods, which are common in implementation research, the analysis was done in the form of a narrative synthesis, which limits generalisability but allows for a richer analysis that is useful for describing implementation outcomes and may be transferrable to other settings [38]. Qualitative, feasibility and pilot studies may not be considered gold-standard research evidence; however, they provide valuable insight into challenges and barriers faced in implementation, which is central to the aims of this review. Specific adaptations made in the early stages of intervention development may provide valuable information for similar programmes in the context of LMIC and the use of NSHWs, as well as in-depth data about the lived experience, opinions and concerns of service users, NSHWs and other stakeholders [18].

The relatively small number of quantitative studies meant that it was not feasible to conduct a meta-analysis. The heterogeneity in study design and lack of rigorous implementation outcome framework used by many of the included studies meant that there were inconsistencies in the way the outcomes were measured and defined. Three studies reported that the intervention being assessed had used measurements that were not validated for the setting and had low internal reliability, which may have been unreflective of local understanding of mental health issues and affected the conclusions drawn [8, 16, 42]. External validity may be low for interventions that were designed specifically for one setting and conclusions drawn from them may not be generalisable to other contexts or service user groups [2, 3, 31, 42]. Only one study discussed implementation costs, and none calculated estimates of the cost in terms of increased penetration. Without cost-effectiveness data, it is difficult to make an argument for scale-up, especially in low-resource settings [8].

This review reinforces the importance of using rigorously defined outcomes [37] to evaluate implementation of NSHW-delivered interventions, in order to better facilitate interpretation and comparisons across studies. We did not aim to draw conclusions about the implementation outcomes of using NSHWs in comparison to specialist mental health care providers, especially given that ‘treatment as usual’ in many contexts was no mental health care. Further, given recent reviews on effectiveness outcomes of NSHW-delivered behavioural interventions in LMICs, the focus of this review was on implementation outcomes as opposed to patient-level effectiveness outcomes.

## Conclusion

This review reported on the feasibility, acceptability and appropriateness of task-sharing CBT-based interventions in LMICs across a range of NSHWs. The findings suggest that CBT may be feasible and acceptable for NSHW delivery, so long as it is appropriately adapted and there is comprehensive training and continual supervision to ensure fidelity of delivery. However, more research is needed to evaluate long-term adoption, widespread penetration, cost and sustainability of implementing NSHW-delivered CBT to address CMD/SUD in LMICs. As issues of implementation will likely be linked to the effectiveness of CBT interventions for particular service user groups, future studies could assess the question of whether, and in what ways, implementation outcomes differ by service user category. There is also a need for a more consistent approach to defining, measuring, reporting and analysing implementation outcome measures, in order to synthesise knowledge about implementation across different studies, to develop task sharing models and to ensure we develop scalable and sustainable approaches to address mental health and substance-use disorders in resource-poor settings globally.

## Appendix

### Search strategy for MEDLINE

1. Low-and Middle-Income Country
2. exp Developing Countr*/
3. (Africa or Asia or Caribbean or West Indies or South America or Latin America or Central America).mp.
4. (Afghanistan or Albania or Algeria or Angola or Antigua or Barbuda or Argentina or Armenia or Armenian or Aruba or Azerbaijan or Bahrain or Bangladesh or Barbados or Benin or Belarus or Byelorussian or Belarus or Belorussian or Belorussia or Belize or Bhutan or Bolivia or Bosnia or Herzegovina or Hercegovina or Botswana or Brazil or Bulgaria or Burkina Faso or Burkina Fasso or Upper Volta or Burundi or Urundi or Cambodia or Khmer Republic or Kampuchea or Cameroon or Cameroons or Cameron or Camerons or Cape Verde or Central African Republic or Chad or Chile or China or Colombia or Comoros or Comoro Islands or Comores or Mayotte or Congo or Zaire or Costa Rica or Cote d’Ivoire or Ivory Coast or Croatia or Cuba or Cyprus or Czechoslovakia or Czech Republic or Slovakia or Slovak Republic or Djibouti or French Somaliland or Dominica or Dominican Republic or East Timor or East Timur or Timor Leste or Ecuador or Egypt or United Arab Republic or El Salvador or Eritrea or Estonia or Ethiopia or Fiji or Gabon or Gabonese Republic or Gambia or Gaza or Georgia Republic or Georgian Republic or Ghana or Gold Coast or Greece or Grenada or Guatemala or Guinea or Guam or Guiana or Guyana or Haiti or Honduras or Hungary or India or Maldives or Indonesia or Iran or
5. or Isle of Man or Jamaica or Jordan or Kazakhstan or Kazakh or Kenya or Kiribati or Korea or Kosovo or Kyrgyzstan or Kirghizia or Kyrgyz Republic or Kirghiz or Kirgizstan or Lao PDR or Laos or Latvia or Lebanon or Lesotho or Basutoland or Liberia or Libya or Lithuania or Macedonia or Madagascar or Malagasy Republic or Malaysia or Malaya or Malay or Sabah or Sarawak or Malawi or Nyasaland or Mali or Malta or Marshall Islands or Mauritania or Mauritius or Agalega Islands or Mexico or Micronesia or Middle East or Moldova or Moldovia or Moldovian or Mongolia or Montenegro or Morocco or Mozambique or Myanmar or Myanma or Burma or Namibia or Nepal or Netherlands Antilles or New Caledonia or Nicaragua or Niger or Nigeria or Northern Mariana Islands or Oman or Muscat or Pakistan or Palau or Palestine or Panama or Paraguay or Peru or Philippines or Philipines or Phillipines or Phillippines or Poland or Portugal or Puerto Rico or Romania or Rumania or Roumania or Russia or Russian or Rwanda or Ruanda or Saint Kitts or St Kitts or Nevis or Saint Lucia or St Lucia or Saint Vincent or St Vincent or Grenadines or Samoa or Samoan Islands or Navigator Island or Navigator Islands or Sao Tome or Saudi Arabia or Senegal or Serbia or Montenegro or Seychelles or Sierra Leone or Slovenia or Sri Lanka or Ceylon or Solomon Islands or Somalia or Sudan or Suriname or Surinam or Swaziland or Syria or Tajikistan or Tadzhikistan or Tadjikistan or Tadzhik or Tanzania or Thailand or Togo or Togolese Republic or Tonga or Trinidad or Tobago or Tunisia or Turkey or Turkmenistan or Turkmen or Uganda or Ukraine or Uruguay or USSR or Soviet Union or Union of Soviet Socialist Republics or Uzbekistan or Uzbek or Vanuatu or New Hebrides or Venezuela or Vietnam or Viet Nam or West Bank or Yemen or Yugoslavia or Zambia or Zimbabwe or Rhodesia).mp.
6. ((developing or least-developed or less-developed or under-developed or underdeveloped or low-income or middle-income or transitional) adj3 (countr* or nation? or world or econom*)).mp.
7. (LAMIC? or LIC? LMIC? or MIC? or UMIC?).mp.
8. ((LAMI or LI or LMI or MI or UMI) adj3 countr*).mp.
9. 1 or 2 or 3 or 4 or 5 or 6
10. Cognitive Behavioural Therapy
11. exp Psychotherapy/
12. exp Counsel?ing/CBT or cognitive behavio?r* therap* or dialectical behavio?r* therap*
13. ((brief or low-intensity or scalable) adj2 (intervention? or treatment?)).mp.
14. ((group or inter-personal or interpersonal or psychosocial or talk* or problem solving or dialectical behavio?r) adj2 (therap* or intervention? or treatment? or program* or package? or training? or approach* or technique*)).mp.
15. (thinking healthy or problem-management or behavio?r* activation or activity scheduling or cognitive restructuring or mindfulness or motivational interviewing or progressive muscle relaxation or diaphragmatic breathing or relapse prevention).mp.
16. ((aversive or aversion or feedback? Or desensiti?ation or relaxation or meditate*) adj3 (therap* or intervention? or program*or treatment* or approach* or technique*)).mp.
17. ((cognition or cognitive) adj3 (therap* or remediation or restructur* or rehabilitat* or intervention* or program* or psychotherapy* or treatment* or approach* or technique*)).mp.
18. ((behavio?r*) adj3 (therap* or remediation or restructur* or rehabilitat* or intervention* or program* or psychotherapy* or treatment* or approach* or technique*)).mp.
19. (CBT or IPT or IPT-G or PST or ACT or DBT or PM or BA).mp.
20. 8 or 9 or 10 or 11 or 12 or 13 or 14 or 15 or 16
21. Non-Specialist Health Workers
22. exp Allied Health Personnel/
23. Community Health Worker? or Nurse* Aide? or Psychiatric Aide? or Caregiver? Or voluntary worker? or volunteer? or Community network? or Social support or Health manpower or barefoot doctor? or Teacher? or School staff or Trainer?.mp.
24. ((community or community based or community-based or lay or voluntary or volunteer? or untrained or trained or unlicen?ed or nonprofessional? or non-professional? or peer? or nonmedical or non-medical or non health or non healthcare) adj3 (worker? or visitor? or attendant? or aide? or support* or person* or helper? or care* or consultant? or advisor? or counsel* or assistant? or staff)).mp.
25. (peer adj (work* or counsel* or deliver*)).mp.
26. ((paraprofessional? or para-professional? or paramedic* or support) adj2 (personnel or worker?)).mp.
27. ((allied health or non-specialist? Or nonspecialist) adj2 (worker? or professional? or personnel)).mp.
28. ((health* or medical* or nurs*) adj3 (auxiliar*)).mp.
29. ((nurs* or psychiatric) adj3 (aid* or assistant* or attendant*)).mp.
30. (informal) adj (care*).mp.
31. ((self-help or support) adj3 (group?)).mp.
32. ((Social or psychosocial) adj (care or support)).mp.
33. (Village adj3 worker?).mp.
34. ((Community or community based or community-based) adj (intervention or health or healthcare or healthcare or service? or network or care or assistance or help or work)).mp.
35. CHW? Or VHW? Or PSW?.mp
36. (task* adj3 (shar* or shift*)).mp.
37. ((collaborative or stepped) adj2 care).mp.
38. (communit*) adj3 (based or intervention* or network* or service?).mp.
39. 18 or 19 or 20 or 21 or 22 or 23 or 24 or 25 or 26 or 27 or 28 or 29 or 30 or 31 or 32 or 33 or 34
40. Mental, Neurological and Substance-Use Disorders
41. (Mental disorders or mental, neurological and substance-use disorders or MNS disorders or common mental disorders or CMD)
42. exp Mental disorders/
43. exp Anxiety disorders/
44. exp Bipolar and related disorders/
45. exp Disruptive, impulsive control and conduct disorders/
46. exp Dissociative disorders/
47. exp Elimination disorders/
48. exp Feeding and eating disorders/
49. exp Mood disorders/
50. Motor disorders.mp.
51. exp Neurocognitive disorders/
52. exp Neurodevelopmental disorders/
53. exp Personality disorders/
54. exp Schizophrenia spectrum and other psychotic disorders/
55. exp Sleep–wake disorders/
56. exp Somatoform disorders/
57. exp Substance-related disorders/
58. exp Trauma and stressor related disorders/
59. 36 or 37 or 38 or 39 or 40 or 41 or 42 or 43 or 44 or 45 or 46 or 47 or 48 or 49 or 50 or 51 or 52 or 53

**(1)** and **(2)** and **(3)** and **(4).**

## Abbreviations

CBT: Cognitive behavioural therapy
CMD: Common mental disorders
ICD-10: International classification of diseases, 10^th^ revision
LMIC: Low- and middle-income countries
MNS: Mental, neurological and substance-use disorders
NSHW: Non-specialist health workers
PTSD: Post-traumatic stress disorder
RCT: Randomised controlled trial
SUD: Substance use disorder
